# Finding the “Sweet Spot”: Sharing the decision-making in ADHD treatment selection

**DOI:** 10.1186/s12991-022-00394-2

**Published:** 2022-05-27

**Authors:** Daniel Tan, Thomas R. King

**Affiliations:** 1 Spectrum Psychiatric Services, Houston, TX USA; 2 Tris Pharma, Inc., 2031 US-130, Monmouth Junction, Monmouth Junction, NJ 08852 USA

**Keywords:** Shared decision-making, ADHD, Stimulant, Amphetamine, Methylphenidate

## Abstract

**Background:**

Stimulants are often prescribed as first-line therapy for attention-deficit/hyperactivity disorder. Currently, there are many therapeutic options available for clinicians and families to consider when making the decision to use a medication. In practice, selection of a stimulant medication for ADHD is highly personalized and can be narrowed down to two major factors: finding the optimal duration of the medication effect, and then estimating a starting dose and subsequently “fine-tuning” the medication to the optimal dosage of the medication. With the possibility of titrating to an optimal stimulant dosage within one prescription of a liquid stimulant, prescribers can recruit the parent/caregiver to actively participate in managing the transition to medication, allowing for greater ownership and a sense of shared control over the process.

**Case presentation:**

The short case series offers a communication method by which clinicians can apply the principles of shared decision-making in helping the parent or caregiver of a newly diagnosed patient with ADHD make informed decisions about medication selection, and to obtain a greater sense of comfort with the new medication regimen.

**Conclusions:**

Much has been published on the importance of clinicians and their patients fostering an environment of clear and unrestricted information-sharing. This short case series illustrates the effectiveness of this approach. Once parents are comfortable with the decision to start drug treatment for ADHD, it is incumbent upon the healthcare provider to ensure that an open channel of communication is maintained, and that parent/caregivers are encouraged to raise concerns as soon as possible.

## Background

Once a child is diagnosed with ADHD, and a decision to proceed with behavioral and/or medication therapy has been reached, the parent/caregiver is faced with a decision-making process that can be bewildering and frightening. There are several marketed formulation options for stimulant and non-stimulant treatments: short-acting, long-acting, morning-dosed, evening-dosed, delayed-release, extended-release, immediate-release, among others. The list of available drug therapies is extensive and can be overwhelming for the parent of a child confronting a new diagnosis of ADHD. Some patients do better with a stimulant, some do not. Each medication has its own safety and tolerability profile that must be considered. Taking all of this into account, it is perfectly understandable that a parent/caregiver may be very concerned when considering medication for their child’s ADHD.

The medical literature provides a wealth of information on the various positive outcomes of medication use to treat ADHD: that it is associated with better academic achievement [1, 2], improvements in family and other social relationships [3], smoother functioning in the early morning, and may provide a multitude of other notable possible improvements ranging from avoidance of later substance abuse [4] and even improvements in overall quality of life [5]. Conversely, each parent/caregiver knows that the decision to use a medication is fraught with drawbacks: the attachment of an ADHD “stigma” to a child by peers and even educators [6]; the need to have a child’s daily routine interrupted (including possibly sleep as well as time spent in school) for administering medication; and the possibility of having to split medication for children in divided family situations. The decision to medicate a child for treatment of ADHD is one that has major impact on both the family as well as the child. Clearly, parents need powerful advocates and information resources to support medication decision-making. Treatment adherence is directly connected to satisfaction with treatment [7]. Once the decision to use a medication has been made, encouragement by the healthcare professional of parent/caregiver involvement in the process is of obvious importance in facilitating a smooth transition to medication use [8].

The use of a stimulant boils down to two major factors: finding the optimal duration of the medication effect, and then estimating a starting dose and subsequently “fine-tuning” the medication to the optimal dosage of the medication. Stimulants have come a long way since the introduction of amphetamine in 1955 and its later adoption as a treatment for ADHD. In recent years, advances in ADHD medication formulations have allowed for once-daily administration with extended durations of effect (in some cases, up to 13 h). Additionally, with the advent of liquid stimulant formulations, precisely targeted dosage plans to find the best possible intersection of efficacy and minimal side effects can be developed through minimal trial and error. For example, the availability of a long-acting liquid form of amphetamine can be titrated in increments as low as 0.5 ml, allowing for up to 16 different dose strengths when taking into consideration the maximum daily dose permitted by the product label.

Prescribers have long sought to increase involvement with bewildered parent/caregivers in the treatment of their child’s ADHD. The observations and reports from parents, other family members, and teachers are of utmost importance in managing a child’s ADHD. Previous stimulant medication regimens required multiple phone calls or visits to the physician, to allow the parent/caregiver an opportunity to share their observations about the drug’s effects (positive and negative), and then to allow to the prescriber to offer an adjustment to the medication dose or possibly a treatment alternative. With the possibility of titrating to an optimal dosage within one prescription of a liquid stimulant, prescribers can recruit the parent/caregiver to actively participate in managing the transition to medication, allowing for greater ownership and a sense of shared control over the process. This requires the close oversight of the healthcare provider, as well as an implicit agreement of trust between both parties to ensure clear and timely communication of any issues arising during the “fine-tuning” process. Here, we offer a practical case history where the parent has adopted some of the responsibility for control of medication adjustment under the guidance of the treating physician.

## Case presentation

### Patient 1: Joey

**Age: 6 years, 9 months**

**Diagnosis: ADHD combined type, severe**

#### Presenting symptoms and complaints

Joey has no prior diagnosis and who just started kindergarten. At the first 6-week progress report, his kindergarten teacher requested a parent–teacher conference because Joey seemed hyperactive and had difficulty with focusing on tasks. There were many times when he would be disengaged from the rest of the class and could not follow directions from the teacher even though the instructions were simple and age appropriate. The kindergarten teacher felt very strongly that his parents arrange for an appropriate evaluation and hence the psychiatric evaluation.

Despite a clear explanation of the diagnosis of ADHD, and a review of treatment options, the parents were especially reluctant and hesitant about the use of psychotropic medications. The parents were naïve about ADHD and the mother admitted to struggling with the notion that their son was somehow “defective” and needed medication intervention. It was at this point that his child psychiatrist suggested that they try a liquid stimulant as it would afford the parents the opportunity to gradually titrate the medication from a very small initial dose to an effective and optimal dose.

### Initial treatment plan

Joey was started on amphetamine extended-release oral suspension and an initial variable dose prescription was written as follows:

**Table:** 

**Amphetamine extended-release oral suspension (amphetamine extended-release oral suspension 2.5 mg/ml)**
**Sig. 1 ml to 4 ml po q am**
**Quantity: #120 ml**

Joey’s parents were given very clear and detailed instructions to try an initial dose of 1 ml every morning for 4 days and to increase/titrate this dose by either ½- or 1-ml increments every 4 days but not to exceed the prescribed limit of 4 ml. The parents were also informed that there was a good likelihood that they will see a positive response somewhere between 2 to 4 ml and to stop at that effective dose once they could detect or notice a positive difference. They were also informed that if they saw a detectable response earlier, perhaps at 2.5 ml, 3 ml, 3.5 ml, or even 4 ml, to remain at that effective dose, instructed not proceed further, and certainly to not dose beyond 4 ml.

#### Follow-up

At the 1-month follow-up appointment, Joey’s parents reported that they followed the instructions carefully and found Joey’s “effective sweet spot dose” at 3.5 ml/day. They were most happy and relieved to report that Joey’s functioning in class had improved considerably and that his kindergarten teacher was pleased with his progress and that he was now able to participate along with the other children in the class. The parents were also grateful that they had been allowed to participate actively in his treatment and that they had been involved in a shared decision-making endeavor for their child in finding the optimal dosage for their child.

### Patient 2: Lisa

**Age: 9 years 3 months**

**Diagnosis: ADHD inattentive type**

#### Presenting symptoms and complaints

Lisa is a 4th grader who was initially diagnosed with ADHD when she was in the 2nd grade. Despite Lisa’s efforts to concentrate and stay focused, she often day-dreamed, could not stay on task and was very easily distracted. Her teachers reported that she frequently performed below her academic potential. Despite having been treated initially by a pediatrician and then later by a child psychiatrist, the mother felt that Lisa had not responded well to any of the medications that had been tried and that they caused unmanageable side effects. Lisa had been tried on more than six different ADHD medications, but with limited benefit. Furthermore, during the initial assessment the mother admitted that she had a general mistrust of stimulants even though she had read that these medications were safe and effective. The mother complained of dissatisfaction with the results so far and was concerned that Lisa was being overmedicated. She confided that she had considered resorting to the use of wellness products and herbal preparations to treat her child’s ADHD.

Following these admissions and disclosures by the mother, it became clear that the treatment failures thus far were related to the mother’s own fears of the use of these medications. Lisa’s psychiatrist suggested that the mother try a liquid stimulant so that the she (the mother) could adjust and customize the dosage and discover for herself the least amount of medication necessary to provide an effective dose to improve her child’s problems with inattention.

#### Initial treatment plan

Since Lisa had last been on a daily morning mid-range (20 mg) dose of dexmethylphenidate HCl, a variable dose prescription of methylphenidate extended-release oral suspension was started as follows:

**Table:** 

**Methylphenidate extended-release oral suspension 5 mg/ml**
**Sig. 4 ml to 8 ml po q am**
**Quantity: 2 × 600 mg (150 ml) bottles**

The mother was advised to start with 1 ml and increase the dosage by ½ or 1 ml increments every 3 to 4 days until she found an effective dose that improved her daughter’s ability to focus and pay attention. The mother was also advised that there was a good likelihood that Lisa would respond somewhere between 6 to 8 ml and that she could stop at any dosage as long as it did not exceed 8 ml.

#### Follow-up

Both Lisa and her mother returned for the 1-month follow-up appointment with a glowing report that Lisa had responded well with 6.5 ml of methylphenidate extended-release oral suspension and that it was an effective dosage point that did not cause any adverse side effects. The mother was most grateful for having been invited to share in the decision-making process of her daughter’s treatment. The mother herself had discovered the “best” dosage for her child while at the same time achieving a high level of comfort by controlling a medication that she had feared and did not trust previously.

## Discussion and conclusion

The “sweet spot” of shared medication decision-making lies on the continuum between complete physician control of medication choice and management to caregiver informed choice [9]. The concept of shared decision-making in choosing any medical treatment is regarded as a best practice by both the American Academy of Pediatrics [10] and the US Department of Health and Human Services [11]. Evidence shows that most primary care pediatricians involve parents in ADHD treatment decision-making [12]. One survey performed indicated that most parents felt most like decision-making partners in the medical care of their children in the setting of a patient-centered medical home [13]. On the other hand, a separate study showed that only 44% of parents of a child with psychosocial problems reported that their child’s physician always asked about parents’ ideas and opinions on the care of their child [14, 15]. An additional exploratory study showed low levels of shared decision-making behaviors in treatment of children with ADHD, and this problem is exacerbated in non-White families, at lower socio-economic status, and with parents of lower educational level [15]. Clearly, there is room for improvement in ensuring involvement of parents in treatment decision-making.

For the healthcare provider, there are several important points to consider relative to the communication process. Dosing tools used in administration of liquid medication may present a barrier to parents with low or limited health literacy [16], providing a possible impediment to adapting flexible dosing strategies. Barriers to access of specialty care and the overall complexity of ADHD are impediments to any treatment success and must be addressed before proceeding [17]. Parent attitudes are an important determining factor in achieving optimal adherence to treatment, and in a shared decision-making model, it behooves the healthcare provider to carefully assess parent suitability at initial evaluation to ensure the chances of success [18]. However, research indicates most families prefer to be the primary or secondary decision-makers in choosing ADHD treatment [19], so HCPs should carefully screen and address any potential issues upfront when assessing parent/caregiver suitability for shared decision-making.

Developing realistic goals with ADHD treatment requires careful, open discussion before initiating treatment. As demonstrated with Lisa’s case, finding the right stimulant at the right dose can be a frustrating and time-consuming process for the patient as well as the parent/caregiver. Patients are known to have variable responses to stimulants. Fostering an environment of open communication between the parent/caregiver and the healthcare provider is essential to ensure that all observations about treatment, whether positive or negative. Once parents are comfortable with the decision to start drug treatment for ADHD, it is incumbent upon the healthcare provider to ensure that an open channel of communication is maintained, and that parent/caregivers are encouraged to raise concerns as soon as possible.

## Data Availability

Not applicable.
